# Human evolution: the non-coding revolution

**DOI:** 10.1186/s12915-017-0428-9

**Published:** 2017-10-02

**Authors:** Lucía F. Franchini, Katherine S. Pollard

**Affiliations:** 1 Instituto de Investigaciones en Ingeniería Genética y Biología Molecular (INGEBI), Consejo Nacional de Investigaciones Científicas y Técnicas (CONICET), Buenos Aires, Argentina; 2 Gladstone Institutes, San Francisco, CA 94158 USA; 3 Department of Epidemiology & Biostatistics, Institute for Human Genetics, Institute for Computational Health Sciences, University of California, San Francisco, CA 94158 USA

## Abstract

What made us human? Gene expression changes clearly played a significant part in human evolution, but pinpointing the causal regulatory mutations is hard. Comparative genomics enabled the identification of human accelerated regions (HARs) and other human-specific genome sequences. The major challenge in the past decade has been to link diverged sequences to uniquely human biology. This review discusses approaches to this problem, progress made at the molecular level, and prospects for moving towards genetic causes for uniquely human biology.

## Post-genomic challenges for determining uniquely human biology

When the human genome was first sequenced [1, 2], the big question was “how many genes do we have?” Most people guessed too high. Sequencing our closest living relative, the chimpanzee [3, 4], begged the question “which genes are different?” Here the answer was predicted a century before and supported by King and Wilson’s 1975 discovery that certain blood proteins have very few amino acid differences between human and chimpanzee [5, 6]. We now know that the vast majority of all genomic changes that happened since the human–chimpanzee ancestor are in non-coding regions, consistent with King and Wilson’s hypothesis that regulatory changes drove the differences between our species. In hindsight, the importance of gene regulation in human evolution is logical. There are many more DNA bases in regulatory regions than in protein-coding genes, making them a larger target for evolutionary innovation. Furthermore, genes frequently function in many different contexts, and this pleiotropy constrains their evolution compared to regulatory elements, which tend to be more modular [7]. Thus, regulatory sequences have great potential to be drivers of human evolution.

The challenge in the post-genomic era has been to determine which of the millions of human-specific non-coding sequence differences are responsible for the unique aspects of our biology. This is a hard problem for many reasons. First, the non-coding genome is vast, requiring methods to prioritize the mutations that matter. The neutral theory of molecular evolution, coupled with redundancy in biological networks, suggests that many human-specific DNA changes had little effect on our biology. Second, we know much less about how sequence determines function of regulatory elements compared to protein or RNA genes. Hence it is difficult to predict the molecular, cellular, and organismal consequences of human-specific regulatory mutations. Furthermore, most uniquely human traits are complex, and there is no doubt that they are encoded by a combination of mutations in different genomic loci. Finally, because gene regulation has diverged significantly between primates and model organisms such as mice, zebrafish or flies, it is hard to test hypotheses about the functional effects of regulatory mutations. In this review, we discuss advances to address these barriers with an emphasis on linking sequence to function, complementing other recent papers that explore genetic and regulatory changes in human evolution [8–13].

## Discovering the fastest evolving regions in the human genome

**Fig. 1.**
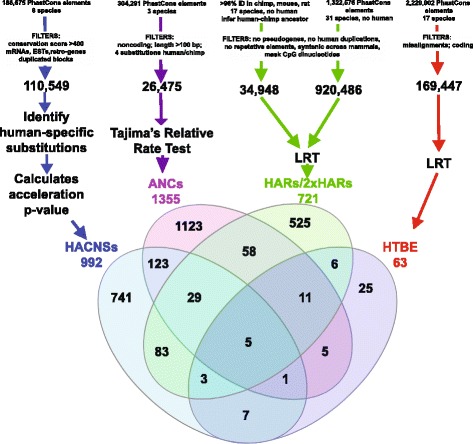
Identification of human accelerated elements. *Top*: the four different approaches used to identify human accelerated regions. Some key differences include (i) the conserved elements used as candidates to identify HARs (which depend on multiple sequence alignments, methods to detect conservation, and whether human was masked in the alignments), (ii) bioinformatics filters that aim to restrict to non-coding elements and/or remove assembly or alignment artifacts, and (iii) tests used to detect acceleration. *Bottom*: overlap of the different datasets of human accelerated regions. *Abbreviations*: *ANC* accelerated conserved non-coding sequences [20]; *HACNS* human accelerated conserved non-coding sequences [23]; *HTBE* human terminal branch elements [21]. HARs include the original HARs [19] and the second generation HARs or 2xHARs [100]

Single nucleotide changes can have functional consequences, but currently these are difficult to predict in non-coding regions where small mutations are frequently tolerated and the function of a particular nucleotide is rarely known. Hence, human evolutionary genetics has mostly focused on genome regions with many human-specific differences (reviewed in [14–17]). Human accelerated regions (HARs) are short, evolutionarily conserved DNA sequences that have acquired significantly more DNA substitutions than expected in the human lineage since divergence from chimpanzees. A number of studies applied different tests to identify HARs either genome-wide [18, 19] or with protein-coding sequences specifically removed from the analysis [20–23]. We and the other authors of these studies had a common aim: to identify regulatory elements with human-specific activity (Fig. 1). These analyses started with regions conserved across non-human mammals in order to enrich for functional elements [24–27] and to increase power to detect acceleration (Box 1). Then they used various methods to identify a subset of conserved elements that accumulated human-specific changes. Differences in analysis choices and available data over time (for example, species in alignments, methods used to identify conserved elements, tests for acceleration, bioinformatics filters to remove artifacts) resulted in only modest overlap between the HARs identified in different studies despite their common aim (Fig. 1). These studies also differed in whether they specifically tested for positive selection compared to a neutral model or simply identified acceleration, which could be due to a variety of evolutionary processes including mutation rate increases or loss of constraint (Box 1). Collectively nearly 3000 non-coding HARs have been identified to date [14], representing a pool of candidates that can be searched for regulatory regions with human-specific activity.

Insertions, deletions, duplications, and rearrangements—collectively known as structural variants (SVs)—contribute many more nucleotides to the genetic difference between humans and chimpanzees than do HARs and other single nucleotide variants. Indeed, the first differences detected between the human and chimp genomes were large SVs discovered prior to genome sequencing (for example, chromosome 2 fusion, inversions on chromosomes 1 and 18 [28]). Primates have accumulated SVs at an accelerated rate compared to other mammals, and the human genome contains numerous lineage-specific SVs [29, 30]. These SVs contribute significantly to the emergence of new genes and gene families (reviewed in [31]). Most HAR analyses have specifically filtered out SVs due to the difficulty of accurately assembling and aligning these regions. But HARs are in fact common in recent segmental duplications [20], and we found them to be enriched near duplicated genes [32]. In addition, SVs can change gene expression and phenotypes by associating non-coding elements with genes they did not previously regulate (‘enhancer hijacking’, see below) [33, 34]. Thus, further exploration of the regulatory consequences of human-specific SVs is clearly needed.

## Beyond conservation: combining acceleration tests and epigenetic marks

Sequence conservation is a useful tool for predicting which non-coding substitutions will be functional. But many regulatory elements are not conserved [35, 36], and conservation does not indicate when and where a regulatory element is active. Since the initial discovery of HARs and human-specific SVs, understanding of the proteins and epigenetic marks found at particular categories of regulatory element has improved significantly, as have genome-wide functional genomics assays such as chromatin immunoprecipitation sequencing (ChIP-seq), ATAC-seq, and RNA-seq. Using these tools it is now possible to generate genome-wide maps of predicted regulatory regions across many cell types and developmental stages. We predicted enhancers in the human genome and tested 29 predictions overlapping HARs with in vivo reporter assays (see below), discovering that many of the previously identified HARs are enhancers active in the developing embryo [37].

**Fig. 2.**
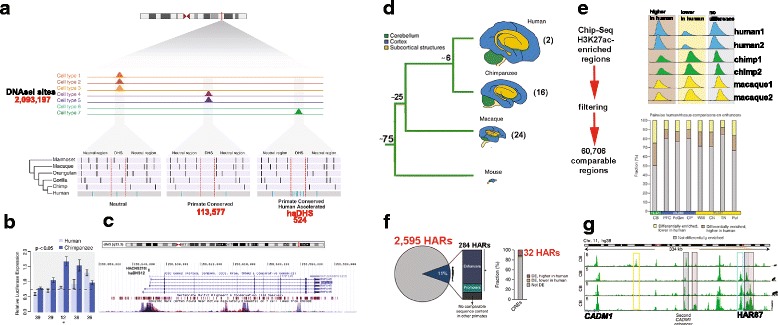
Strategies to identify human-specific enhancers. **a** Methodology used to identify human-accelerated DNSase I hypersensitive sites (haDHSs) in [38]. *Black bars*, nucleotides that differ from the human sequence; *blue bars*, sites where all species differ from humans; *dotted red lines*, DHS and neutral sequence. **b** Five haDHSs showed different activity with human versus chimpanzee sequences in SK-N-MC cells. **c** Differentially active haDHS12/DAR12 (*asterisk*) overlaps previously identified HACNS219 [23] and is located near *RNF145,* a ring finger gene involved in cellular cholesterol metabolism [101]. **d** Brains of species studied in [43] (approximately to scale, colors label regions). Tree indicates approximate timing of splits between lineages (millions of years ago). **e** Workflow and (bottom) fraction of H3K27ac peaks that is differentially enriched (*DE*) between species per brain region. **f** *Left*: Percentage of HARs within conserved regulatory elements (CREs). *Right*: Most of the 240 HAR-containing CREs that align across species were not DE in human versus macaque and chimpanzee. **g** H3K27ac tracks for mouse, macaque, chimpanzee, and human cerebellum. *Gray*, shared enhancers; *purple*, DE enhancers higher in human versus macaque; *red*, *HAR87*. Enhancers gained in mouse or lost in primates (*yellow*) may compensate for the enhancer gained in primates (*light blue*) upstream of the *CADM1* gene. *Abbreviations*: *CB* cerebellum, *PFC* prefrontal cortex, *PcGm* precentral gyrus, *OP* occipital pole, *WM* white matter, *CN* caudate nucleus, *TN* thalamic nucleus, *Put* putamen. Reproduced partially from [38, 43], with permission

Functional genomics data are also very useful for loosening or omitting the requirement that HARs be conserved in other species. Two recent investigations performed evolutionary analyses of putative human regulatory elements and identified many examples of human sequence acceleration missed by studies that relied on deep sequence conservation (for example, greater than 95% sequence identity across mammals or vertebrate phastCons elements). Gittelman et al. performed comprehensive evolutionary and population genetics analyses on open chromatin (specifically, DNase I hypersensitive sites (DHSs)) from 130 cell types to discover regulatory DNA with evidence of adaptive evolution in humans [38]. They discovered 524 DHSs with sequences that are conserved in non-human primates but show accelerated nucleotide substitution rates in the human lineage (haDHS). By functionally characterizing selected haDHSs using transgenic reporters and luciferase assays in different cell types, the authors identified several where the human sequence changes result in a gain of enhancer function (Table 1; Fig. 2).

**Table 1:** Selected examples of human-specific regulatory elements tested in functional assays that showed gain or loss of function

HAR	Genome Location (hg19)	Closest gene	Experimental approach	Biological function	Domain of expression	Reported functional difference	Postulated phenotypic association	Reference
HAR1	chr20:61,733,447–61,733,630	*HAR1F; HAR1R*	qPCR; in situ hybridization	RNA gene	Cajal-Retzius neurons	RNA secondary structure	Brain development	[18]
HAR2/HACNS1	chr2:236773664–236774209	AGAP1 (CENTG2); GBX2	Transgenic mice	Enhancer	Limb, branchial arches	Gain of function	Limb development	[59]
2xHAR.142	chr14:34048651–34048815	*NPAS3*	Transgenic mice	Enhancer	Forebrain, midbrain, spinal cord	Gain of function	Brain development	[57]
HAR202	chr14:34045570–34045772	*NPAS3*	Transgenic zebrafish	Enhancer	Central nervous system	Loss of function	Brain development	[58]
ANC516	chr10:36238421–36239039	*FZD8*	Transgenic mice	Enhancer	Forebrain, midbrain, spinal cord	Gain of function	Brain development	[56]
2xHAR.238	chr2:121832784–121833140	*GLI2*, *TFCP2L1*	Transgenic mice	Enhancer	Forebrain, midbrain, hindbrain and spinal cord	Loss of function	Brain development	[37]
2xHAR.114	chr20:30425200–30425393	*MYLK2, FOXS1*	Transgenic mice	Enhancer	Limb and spinal cord	Loss of function	Limb development	[37]
2xHAR.164	chr2:133346894–133347089	*ANKRD30BL*, GPR39, LYPD1, NCKAP5	Transgenic mice	Enhancer	Forebrain, midbrain, hindbrain, spinal cord	Gain of function	Brain development	[37]
2xHAR.170	chr5:153640309–153640363	GALNT10, SAP30L, HAND1	Transgenic mice	Enhancer	Isthmus and spinal cord	Loss and gain of function	Brain development	[37]
HAR25	chr4:182271922–182272882	*ODZ3*	Transgenic mice	Enhancer	Eye	Loss of function	Eye development	[37]
ANC705	chr15:61,979,891–61,980,090	*VPS13C, RORA*	Epigenetic marks	Enhancer	Neural crest	Gain of function	Neural crest development	[44]
HAR89; 2xHAR.305	chr1:3,089,780–3,089,980	*PRDM16*	Epigenetic marks	Enhancer	Neural crest	Gain of function (chimp)	Neural crest development	[44]
2xHAR.236	chr1:53,992,480–53,992,679	*GLIS1*	Epigenetic marks	ENHANCER	Neural crest	Gain of function (chimp)	Neural crest development	[44]
HACNS825	chr2:217,728,749–217,728,948	*TNP1; LOC105373876*	Epigenetic marks	ENHANCER	Neural crest	Gain of function (chimp)	Neural crest development	[44]
2xHAR87	chr11:115648183–115648526	*CADM1*	Epigenetic marks and 4-C	ENHANCER	Cerebellum	Gain of function compared to macaque	Synapse regulation	[43]
DAR-12/HACNS219	chr5:158227696–158229500	*RNF145*	Luciferase	ENHANCER	SK-N-MC cells	Gain of function	Hematological traits	[38]

Using a similar approach, Dong et al. compared substitution rates of DHSs to nearby ancestral repeats (LINE1 or LINE2 elements, assumed to be neutrally evolving) and identified 3538 accelerated DHSs (ace-DHS) [39]. Notably, only 17 accelerated DHSs overlap between these two studies, likely due to Gittelman et al. requiring conservation in primates so that they tested only 113,577 DHSs versus 808,943 in Dong et al.’s analysis, which had no such conservation filter. In addition, these two approaches used different data to estimate neutral rates for their acceleration tests. Regardless, it is clear that starting with DHSs rather than conserved elements reveals novel human-specific regulatory elements: the majority of haDHSs (454/524) and ace-DHSs (3520/3538) were not previously identified as HARs. It is important, however, to validate the regulatory functions of DHSs and other biochemically active genomic regions experimentally, especially if they are not conserved [36].

## Comparative identification of regulatory regions

An alternative approach to identify human-specific regulatory elements is to perform functional genomics assays such as open chromatin or protein binding in multiple species and find regions with differential evidence of regulatory activity. Some of the first evidence that human-specific regulatory elements may be common came from the ENCODE project, which found that a high proportion (~50%) of the biochemically active regions identified in the human genome were not conserved across mammals [35, 40]. Several recent studies built on this observation and compared human epigenomic profiles (such as open chromatin, histone modifications) to those of primates and other species using either primary tissues or cell lines from various developmental stages (see below). To infer that regulatory marks are human-specific requires epigenomic profiles from chimpanzees (or bonobos), which is often impossible due to limited availability of material and ethical considerations. One potential solution is to investigate post hoc if the regions with epigenetic signatures that differ between human and rodents or monkeys intersect with HARs or harbor sequence changes that are unique to the human genome.

Cotney and colleagues performed ChIP-seq for histone H3 lysine 27 acetylation (H3K27ac, a mark of active enhancers) in human, rhesus macaque, and mouse embryonic limb at four different developmental stages from bud to digit separation, defined as orthologous based on morphology and HOXD gene expression [41]. They found 2175 promoters and 2915 putative enhancers with significantly higher H3K27ac signal in human at one or more time points. Sixteen of these regions overlap with HARs, but most show no evidence of human accelerated substitution rates or unusual diversity patterns across modern humans [41].

Using a similar approach, Reilly et al. mapped active promoters and enhancers using di-methylation of histone H2 lysine 27 (H3K27me2, a promoter mark) and H3K27ac, respectively, during three stages of human, rhesus macaque, and mouse cortex development [42]. Comparing signal for these epigenetic marks across species, the authors predicted 2855 promoters and 8996 enhancers unique to human samples (that is, with higher signal in human than other species). The majority of these regions show no evidence of human accelerated substitution rates, although 48 of them overlap HARs [42].

Vermunt and colleagues assayed H3K27ac in eight anatomical subdivisions of the adult brain (cerebellum, caudate nucleus, thalamic nuclei, putamen, white matter, precentral gyrus, prefrontal cortex, occipital pole) of human, chimpanzee and rhesus macaque [43]. The authors found that 14–43% of putative enhancers and 3–10% of promoters had differential signal between human and macaque brain regions. However, only a very small fraction of these showed a similar difference between human and chimpanzee. While 284 H3K27ac regions from this study overlap with HARs, only 32 of these have significantly higher or lower H3K27ac signal in human versus chimpanzee or macaque. One particularly interesting example is 2xHAR87/HACNS548, which interacts with the promoter of the *CADM1* gene and shows steadily increasing H3K27ac signal from macaque to human (Fig. 2).

Prescott et al. compared transcription factor (TF) and co-activator binding, histone modifications, and chromatin accessibility genome-wide in human and chimpanzee cranial neural crest cells (CNCCs) to pinpoint putative enhancers [44]. They derived CNCCs from pluripotent stem cells using an in vitro protocol in which specification, migration, and maturation are recapitulated in the dish [45, 46], an approach that allows unavailable chimpanzee cell types to be derived in the lab. The authors predicted ~ 1800 regulatory elements with different levels of H3K27ac in human versus chimpanzee, suggestive of differential enhancer activity. A few of these overlap HARs (three of the top 1000; Table 1). The authors found that the variance in H3K27ac signal scales proportionally with human–chimp sequence divergence. We note that sequence differences are quite low overall (three to six substitutions per 500 bp) even at regions with the biggest human–chimpanzee differences in H3K27ac binding [44]. These results show that regions with one or two sequence changes can have different enhancer activity (measured by transgenic assays using luciferase or lacZ), which may make identifying causal sequence changes easier than in HARs with five to ten changes. Prescott et al. speculate that the enhancer activity differences they detect are due to changes in consensus transcription factor binding motifs, such as one called Coordinator. However, since enhancer assays are not highly quantitative (see below) and cannot identify upstream causes of differences in reporter gene activity, further functional experiments are important to validate the expression differences and test the hypothesis that transcription factor binding changes played a causal role in these regions.

**Fig. 3.**
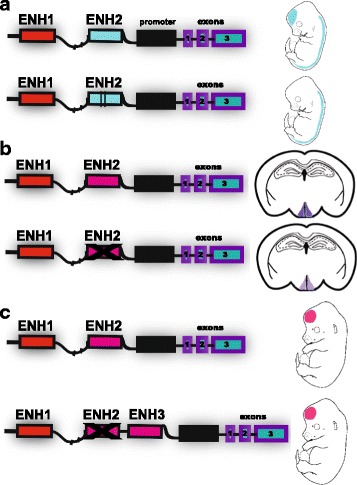
Evolutionary mechanisms at the level of gene regulatory regions. **a** An example of accelerated sequence evolution affecting one enhancer in a locus and leading to gain of an expression domain in the developing forebrain. We characterized one such gain of function HAR in Kamm et al. [57]. **b** Shadow enhancers are multiple enhancers that direct a similar gene expression pattern and thus overlap in function. As such, they can act cooperatively to confer robustness in different physiological situations [77, 78]. The example shown is based on the work of Lam et al. [78], where two enhancers direct expression to the Arcuate Nucleus and both must be deleted in mice to produce a dramatic change in expression and phenotype. **c** Enhancer turnover is when one enhancer disappears and a new one appears in the same regulatory region, replacing the lost function. In the example shown by Domené et al. [72], turnover resulted in no net change in expression or phenotype

Overall, these studies discovered many genome sequences with human-specific epigenomic signatures, which are exciting catalogs of putative regulatory elements for future studies. Since many of these regions are not deeply conserved across mammals, they complement previously identified HARs. However, most of the elements show little or no evidence of human-specific sequence change, suggesting that the causal mutations are in *trans* or that the catalogs contain many false positives due to the many challenges of cross-species functional genomics, such as antibody affinity, low samples sizes, and matching cell composition and developmental stage. As Prescott et al. note, regions with fewer sequence changes than a typical HAR can show enhancer activity differences [44], though we emphasize that it is challenging to discriminate these from the many cases where an equivalent number of sequence changes causes no activity difference. More functional validation of predicted differences in regulatory activity (for example, with reporter assays or CRISPR/Cas9 screens) will help to determine the false positive rate. Meanwhile, we should be careful when interpreting chromatin immunoprecipitation data since protein–DNA binding events cannot be directly translated into regulatory function, as has been discussed elsewhere [47–49]. Another challenge is evaluating the net effect of all genetic and epigenetic changes within a locus on species-specific gene expression (reviewed in [49]), which is particularly difficult when regulatory elements function synergistically or redundantly (Fig. 3 and below).

## Transgenic approaches to study the functional impact of non-coding evolution

One approach to investigating the functional effects of human-specific non-coding sequences is to introduce them into model organisms. Despite differences in anatomy and genetic networks across species [50], transgenic and mutant mouse models have been useful for characterizing human-specific genes, such as *ARHGAP11B* [51] and *SRGAP2C* [52], and human-specific gene variants, such as *FOXP2* [53, 54]. Predicted enhancers can also be validated and tested for effects of sequence differences using transient or stable reporter assays in mouse and zebrafish. In general, in transient transgenic enhancer assays (where the F0 generation is analyzed) enhancer function can be evaluated in only one or a small number of embryonic stages, and not in adult tissues. Although we found that many HARs appear to function during development [10], a substantial fraction likely cannot be characterized with this approach. Generation of stable transgenic lines (where the F1 generation is analyzed) could facilitate the analysis of many more developmental stages and adult expression. However, the low throughput of transgenic experiments has limited their utility for characterizing large catalogs of human-specific regulatory elements.

Despite these caveats, a number of human-specific non-coding sequences have been functionally characterized as enhancers in vivo with reporter assays in transgenic animal models. These include two conserved elements deleted in humans (hCONDELs) [55], nearly 70 HARs (summarized in [10]), including nine haDHSs, and an additional seven haDHSs that do not overlap HARs [38]. Reporter assays in transgenic animals can also be used to test the hypothesis that human-specific mutations in non-coding elements altered their enhancer activity, and indeed several HARs and haDHSs show expression differences between constructs carrying the chimpanzee versus human sequence [37, 56–59] (Table 1). It is important to note that transient transgenics can capture gains and losses of enhancer activity in specific tissues or cell types (expression patterns), but they are not quantitative (expression level depends on number and location of random genomic integrations) and hence are unable to capture changes in activity levels. These studies aiming to analyze the function of human-specific non-coding sequences and then to study them comparatively with transgenic model organism enhancer assays are adding valuable information about the functional impact of human-specific DNA changes. Looking ahead, it will be important to supplement these with additional approaches to link enhancer activity differences to gene expression and phenotypes.

Massively parallel reporter assays (MPRAs) show promise in cell lines and may become practical in whole animals [60–62]. In this technique, a library of candidate regulatory enhancer DNA sequences is cloned into a reporter construct containing a unique DNA barcode that will be transcribed if the enhancer is active. RNA-sequencing of the DNA barcodes enables quantitative measurements of enhancer activity, including effects of individual nucleotide variants [62]. Two potential limitations of this technique are length of the candidate enhancers (<200 bp with current DNA synthesis methods) and the fact that they are usually evaluated outside of their genome context. Knock-in strategies where regulatory element variants are replaced in the analyzed locus (such as via CRISPR/Cas9 genome editing) could address this issue. Low throughput transgenic and mutant model animals can complement both techniques. A combination of methods can together enable tests of hypotheses about the effects of human-specific variants on molecular and organismal phenotypes.

## Human variation links accelerated regions to phenotypes

Another way to associate human-specific non-coding regions with traits is to investigate associations between any polymorphisms they harbor and phenotypic variability in humans. The polymorphic sites will be relatively recent mutations that are largely distinct from differences between human and chimpanzee reference genomes. But they may nonetheless shed light on the general function of the accelerated regions in which they occur. For example, gene copy number variants in the human-specific pericentric inversion of chromosome 1 have been associated with human developmental and neurogenetic diseases [15, 52, 63–65], and we showed that HARs that are conserved in primates but not across all mammals are associated with schizophrenia [66]. As many more human genomes are sequenced, population genetic variation in and near to HARs will also help researchers to test hypotheses about the evolutionary forces that created and maintain HARs (Box 1, Fig. 3).

**Fig. 4:**
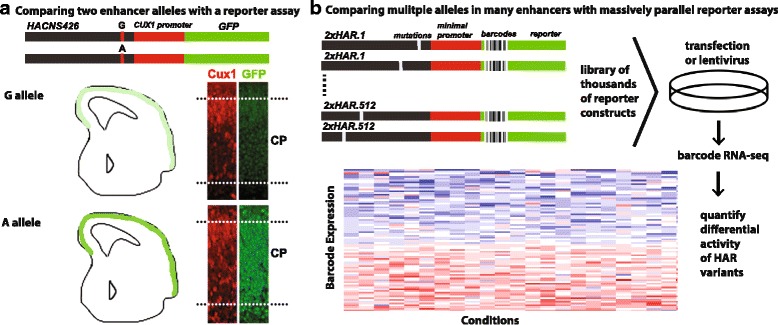
Testing HARs and HAR sequence variants for enhancer activity with reporter assays. **a** Example of a transient transgenic reporter assay to test a HAR (HACNS426) for enhancer activity in mouse embryos. The experiment compares enhancer activity of the major allele (G) to that of the autism-associated minor allele (A) that is never homozygous in healthy controls and is predicted to change transcription factor binding. *Top*: constructs carrying each of the two HACNS426 alleles fused to the human *CUX1* promoter and the GFP reporter gene are separately injected into single-cell mouse embryos. *Bottom*: to assay enhancer activity, brain slices from embryonic day E16.5 are stained for GFP. The authors observed differences in GFP expression with the G allele (*above*) versus A allele (*below*). The major strength of the approach is the spatial and cellular resolution of in vivo measurements, while weaknesses include not being highly quantitative, the use of mouse to compare human and chimpanzee variants, low throughput, and relatively high cost. Adapted with permission from [23]. The study also performed in vitro luciferase reporter assays and showed HANCS426 interacts with the dosage-sensitive *CUX1* promoter. **b** Massively parallel reporter assays (MPRAs) enable thousands of reporter constructs to be tested as a library (*top*) in which each HAR variant is associated with a unique DNA barcode (such as 20-bp sequence). RNA sequencing of barcodes (*bottom*) provides a quantitative estimate of the activity of each HAR variant. MPRAs are high throughput, allowing thousands of HARs and variants thereof to be tested, and they are quantitative, enabling detection of single nucleotide differences with moderate effects on expression. Current weaknesses of the technology include being limited to HARs or HAR segments less than 200 bp and being restricted to testing in cell lines or mouse tail vein assays

Leveraging this approach, Doan et al. sequenced HARs in individuals with autism spectrum disorder (ASD) and unaffected controls [60]. ASD was associated with a significant 43% excess of rare biallelic variants in HARs. The authors showed that 29% of these variants alter enhancer activity in primary mouse neurospheres using a custom MPRA. They further identified rare homozygous mutations with active regulatory marks near neurodevelopmental and disease-associated genes in ASD patients who lack causal coding mutations. Using luciferase reporter assays and transgenic mice, the authors show that one particular ASD-associated HAR variant (HACNS426) that was previously shown to interact with the neuronal morphology gene *Cux1* [67–70] is an enhancer (Fig. 4). The authors report that transgenic mice carrying the mutant allele fused to the *CUX1* promoter and to GFP show elevated expression of the reporter gene in the developing brain compared to the wild-type allele. But it is important to remember that transgenic enhancer assays cannot capture quantitative expression differences (see above).

The study of Doan et al. demonstrates the potential for human sequencing studies coupled with deep phenotyping to shed light on HAR function. Using associations between specific HARs and ASD, the authors conclude that many HARs are essential for normal development. Even though the ASD-associated mutations are different from the substitutions that happened during human evolution, they nonetheless help to annotate the function of the genomic region containing a HAR. Polymorphic deletions of HARs could similarly be used to annotate their functions. Doan et al. further hypothesize that human-specific mutations in HARs could have altered social and/or cognitive behavior. To test this hypothesis precisely with medical re-sequencing requires that specific human–chimp differences and combinations thereof be polymorphic in living humans or primates. As targeted sequencing (for example, with molecular inversion probes [71]) enables HARs to be sequenced in millions of individuals, it will become clear how many human–chimp differences are present in modern humans. One caveat is that the genomes of modern humans and other primates differ from those of ancestral populations, so phenotype associations of HAR mutations must be interpreted in the context of the modern genetic background. For this approach to be a useful path to elucidating HAR functions in humans, it will also be important to couple sequencing data with a very wide range of reliably measured phenotypes ranging from diseases to behaviors. This is an exciting and increasingly feasible direction.

## Changing things so everything stays the same? Regulatory element turnover and compensatory evolution

To measure the effects of non-coding mutations on expression and phenotypes, we must move towards considering the whole regulatory region of a gene, as well as changes in *trans* (such as, expression and activity of upstream regulators). One reason for this is that regulatory elements without sufficient sequence similarity to be aligned across species can direct conserved expression patterns, a phenomenon known as ‘enhancer turnover’ [72–74] (Fig. 3). Another consideration is the fact that regulatory elements interact with each other to drive gene expression, which means that non-coding mutations can amplify or buffer one another even if they are not adjacent. One particular type of compensation across regulatory elements involves ‘shadow enhancers’, which are distinct sequences capable of guiding similar spatiotemporal expression patterns [75–80] (Fig. 3). This apparently redundant function may ensure robust, precise, and stable regulation [77, 78]. Thus, gene expression evolution cannot be easily predicted from a single human-specific regulatory element without evaluating the complete regulatory landscape of the locus.

Most human-specific regulatory elements have nonetheless been characterized one at a time, or even one mutation at a time. Supporting the idea that this limits our ability to predict expression divergence, Vermunt et al. showed that many loci contain both gains and losses of predicted enhancers (4.9–10.3% across brain regions of mice, macaques, chimpanzees, and humans) [43]. For example, the locus containing HAR87 harbors multiple other predicted enhancers with increased or decreased activity during human evolution, measured via chromatin capture coverage (Fig. 2). These other changes could compensate for or otherwise interact with changes in the activity of HAR87. To investigate this possibility, comparative genomics should be combined with functional genomics assays, especially those that do not depend on antibody affinities (for example, chromatin capture, open chromatin).

An emerging approach is to leverage high-throughput genetic screens that can probe combinations of mutations across a locus, for example, via CRISPR genome editing [81, 82]. Using a combination of CRISPR activation and inhibition, as well as knock-outs and knock-ins, it will be possible to study the effect of human–chimpanzee non-coding sequence differences on gene expression in cells or in models such as mice. These studies should investigate all the putative regulatory regions of a gene individually and in combination to truly decipher the net regulatory effects.

## Thinking again: what are we missing in the study of genetic bases of human evolution?

The past decade has seen significant progress towards addressing the major hurdles of associating human-specific non-coding elements with traits that make our species unique. Functional genomics has been transformative in terms of annotating and prioritizing HARs, and it has also been used directly to identify novel human-specific regulatory elements that are not divergent enough in sequence to be detectable in genome-wide tests for accelerated sequence evolution. As the mechanisms of gene regulation are increasingly understood, the functional effects of human-specific non-coding mutations are becoming less mysterious. High-throughput functional assays, such as massively parallel reporter assays and CRISPR screens, are increasing the numbers of characterized human-specific regulatory elements by orders of magnitude. Meanwhile, targeted and whole genome sequencing of thousands of people is enabling rare variant association and linkage studies that connect non-coding elements to traits and may even reveal the phenotypes of individuals carrying specific ancestral versus derived haplotypes. Human evolutionary studies will always be challenged by our inability to test specific hypotheses about genetic changes in the correct organismal context (an ancestral human). However, we now have a much broader collection of complementary research tools to address this problem, including on one hand genome editing of stem cell-derived cell lines, organoids, human primary cells, and model organisms, and on the other hand deep phenotyping and sequencing of humans and other primates to capture natural variation.

Despite these exciting advances towards understanding the role of regulatory changes in human evolution, it is important to ask what hurdles still remain. One major barrier is our very limited ability to assay functions of regulatory elements other than enhancers. Genome editing is helping to address this bias by enabling researchers to introduce individual human mutations or to knock out single human-specific elements [81, 82]. By performing these experiments in primate cells, human cells, and model organisms, it will be feasible to characterize putative human-specific insulating and repressing elements in terms of their downstream effects on molecular and cellular phenotypes. The development of new high-throughput assays to read out regulatory functions other than enhancing gene expression (such as repression or insulation) will help as well.

Another missing piece in the story of human regulatory evolution is the vast sequence differences encoded within SVs, many of which are missing or incorrect in human and non-human primate reference genomes [83]. Even within the SVs that have been accurately compared across primates, human non-coding evolution is relatively unexplored, due largely to the technical challenges of functionally testing regions with duplications and complex genomic architectures. SVs have immense potential to alter regulatory elements and their interactions with target genes. For example, inserting or deleting topologically associating domain (TAD) boundaries, which are often conserved across cell types and species [84], can associate regulatory elements with new genes (termed ‘enhancer hijacking’) or insulate them from their ancestral gene targets [85, 86]. It will be exciting to explore the evolution of human gene expression in terms of 3D genome organization and its effects on regulatory interactions.

## Outlook: what will it take to crack the code?

The next decade could be the one in which the regulatory code is cracked, opening the door to reading out the functional effects of non-coding changes that distinguish humans from other primates. To realize this goal, we should not only push to characterize the human–chimp differences for which we already have functional hypotheses. We must also continue to ask ‘what genomic regions are missing from our analyses?’ and ‘where do current models of gene regulation fail to explain divergently expressed genes?’

## **Box 1** Evolutionary forces underlying the appearance of HARs

In order to detect acceleration in a particular lineage it is necessary to statistically test for a difference between the substitution rate observed on that lineage and the expected rate given the rest of the tree. The tests to detect acceleration typically use continuous time Markov models of DNA (or protein) evolution to quantify the likelihood of the multiple sequence alignment for a genome region [87]. Comparing human to chimpanzee and other primates, acceleration tests can reach genome-wide significance even when there are only a small number of human-specific substitutions in a ~ 100-bp region. This is especially true in regions that are conserved across non-human primates, because the number of expected substitutions is close to zero [14]

Is acceleration equal to positive selection? Not necessarily. In order to differentiate positive selection from relaxation of constraint (both mechanisms can produce acceleration) it is important to compare the evolutionary rate of the accelerated element to the local neutral rate. Using this approach, it has been shown that many HARs (~50%) and haDHSs (~90%) are evolving faster than the neutral rate for that part of the genome, which is suggestive of adaptive evolution [88]. These divergence-based tests will detect fixed differences that largely accumulated millions of years ago. To test for recent or ongoing positive selection, one uses population genetics tests that detect reduced polymorphism relative to divergence or long haplotypes. While many sequence changes in HARs predate diversification of modern humans, some HARs do show evidence for recent selection [19, 21, 88], as do some haDHSs [38]. Another mechanism that likely shaped ~ 20% of HARs is GC-biased gene conversion [88–90], a neutral recombination-associated process that leads to accelerated fixation of weak to strong (AT to GC) mutations. Another ~ 20% of HARs are not evolving faster than neutral expectations, but only faster than expected given strong conservation in other species, perhaps due to loss of functional constraint. Thus, there is not a unique underlying evolutionary mechanism that led to the appearance of all HARs. The interpretation of functional differences between orthologous sequences in HARs should consider different evolutionary forces.

## **Box 2** Our place on earth: humans as primates

When Linnaeus (1758) described our species, he named us *Homo sapiens* and placed us along with orangutans and chimpanzees in the genus *Homo* like any other primate, even though he believed that humans were special beings in God’s creation. Since then diverse data (bones, embryology, fossils, DNA) have verified our place on the tree of life, while also revealing numerous differences between humans and other primates, particularly our closest living relatives the chimpanzees. These include distinct behaviors, morphological characteristics, and molecular phenotypes [91, 92] (see also http://carta.anthropogeny.org/content/about-moca). The uniqueness of the human brain has been investigated in particular detail. Although it is a very typical primate brain overall [16, 93–95], the human brain seems to be unusual in terms of the number of neurons [93, 96], spatial organization [97], neuropil space, amount of dendritic branching, and synaptic spine density [98, 99]
